# A Daylength Recognition Model of Photoperiodic Flowering

**DOI:** 10.3389/fpls.2021.778515

**Published:** 2021-11-18

**Authors:** Xiaoying Wang, Peng Zhou, Rongyu Huang, Jianfu Zhang, Xinhao Ouyang

**Affiliations:** ^1^State Key Laboratory of Cellular Stress Biology, School of Life Sciences, Xiamen University, Xiamen, China; ^2^Rice Research Institute, Fujian Academy of Agricultural Sciences, Fuzhou, China

**Keywords:** photoperiodic flowering, daylength sensing, latitude adaptation, florigen, photoperiod model

## Abstract

The photoperiodic flowering pathway is crucial for plant development to synchronize internal signaling events and external seasons. One hundred years after photoperiodic flowering was discovered, the underlying core signaling network has been elucidated in model plants such as Arabidopsis (*Arabidopsis thaliana*), rice (*Oryza sativa*), and soybean (*Glycine max*). Here, we review the progress made in the photoperiodic flowering area and summarize previously accepted photoperiodic flowering models. We then introduce a new model based on daylength recognition by florigen. By determining the expression levels of the florigen gene, this model can assess the mechanism of daylength sensing and crop latitude adaptation. Future applications of this model under the constraints of global climate change are discussed.

## Introduction

Light is an essential environmental factor regulating plant growth and development, including seed germination, photomorphogenesis, shade avoidance, and photoperiodic flowering. Photoperiodic flowering, which synchronizes flowering time and external seasons based on daylength, is significant for environmental adaptation and plant reproduction. It has been more than 100years since the discovery of photoperiodic flowering. Over the decades, several landmark research breakthroughs, including the study of the tobacco (*Nicotiana tabacum*) cultivar “Maryland Mammoth,” the identification of florigen, the study and understanding of night breaks, the dissection of the molecular mechanisms of flowering in Arabidopsis, and research focused on crop latitude adaptation, have led to a comprehensive model of the molecular mechanisms of photoperiod flowering and revealed breeding applications. In this article, we review the developmental processes and landmark events that have contributed to and shaped the photoperiodic flowering field.

## Discovery of Photoperiodic Flowering in Plants

In Henfrey (1852) suggested that the daylength at different latitudes in summer might be responsible for the natural distribution of plants (Henfrey, 1852). In the nineteenth century, the invention of light bulbs made it possible to expose plants to light at different times, which was exploited by some horticulturists to promote the flowering of summer plants by extending light duration during other seasons. Between 1912 and 1914, Julien Tournois worked on hops (*Humulus* sp.) and *Cannabis* [short-day plants (SDP)] and concluded that shorter daylength can accelerate their flowering (Tournois, 1914). Conversely, Hans Klebs determined that extended daylength can accelerate flowering of cobweb sedum [*Sempervivum funkii*, a long-day plant (LDP)] during winter (Klebs, 1913). In Garner and Allard (1920) formally proposed the photoperiod flowering phenomenon, based on their study of Maryland Mammoth (a tobacco cultivar that continues to grow outdoors in the summer without flowering). They observed Maryland Mammoth growing under natural summer conditions and moved plants to a ventilated darkroom during the afternoon to induce flowering. They suggested that shortening daylength can induce flowering in tobacco (Garner and Allard, 1920). In Garner and Allard (1922) named this phenomenon “photoperiodism.” After proposing the concept of photoperiodism, plant physiologists classified plants into three categories according to their response to photoperiod (Thomas and Vince-Prue, 1996). LDPs only flower when exposed to longer days, while SDP flower only under short days. Day-neutral plants can flower under any daylength, and their flowering is not strictly related to daylength.

## Discovery of Florigen

Leaves have long been recognized as the major plant tissue responsible for sensing daylength. Knott first demonstrated this point in the LDP spinach (*Spinacia oleracea*): Exposing the leaves to long days (LDs) resulted in the initiation of floral primordia at the shoot apex, but plants remained vegetative when the shoot apex alone was treated (Knott, 1934). Zeevaart observed that grafting donor leaves exposed to short days (SDs) from the SDP *Perilla crispa* and *Xanthium pensylvanicum* onto receptor plants of the same species resulted in flowering of the receptor plants growing under LDs (Zeevaart, 1958). Such experiments supported the hypothesis that an unknown factor must be produced in leaves and transported to the shoot apical meristem (SAM), where it promotes flowering. Thus, the concept of “florigen” was introduced to represent this flowering hormone. The botanist Chailakhyan hypothesized that florigen is produced in leaves induced by photoperiod and transported to the shoot apex to induce flowering (Chailakhyan, 1936). However, the florigen molecule itself was not identified until 2005, when scientists cloned the gene *FLOWERING LOCUS T* (*FT*). *FT* encodes a member of the phosphatidyl ethylamine binding protein (PEBP) family that is produced in the vascular leaf bundle and transferred from the phloem to the shoot apex to induce the floral transition (Kardailsky et al., 1999; Corbesier et al., 2007; Tamaki et al., 2007; Notaguchi et al., 2008; Komiya et al., 2009). Once florigen enters shoot apical cells, it first binds to a 14-3-3 protein in the cytoplasm, forming a complex that enters the nucleus and interacts with the basic leucine zipper transcription factor FD to form a florigen activation complex (FAC). The FAC then induces the expression of floral meristem identity genes such as *APETALA 1* (*AP1*), leading to floral induction (Taoka et al., 2011).

Florigen is ubiquitous in flowering plants, but the number of *FT* homologues varies among plants. For example, rice (*Oryza sativa*) harbors 13 *FT* homologous genes (Izawa et al., 2002; Kojima et al., 2002; Komiya et al., 2008). The maize (*Zea mays*) genome encodes 15 *FT* homologues (Danilevskaya et al., 2008; Meng et al., 2011). Soybean (*Glycine max*) counts 10 *FT* homologues (Kong et al., 2010). Besides rice *HEADING DATE 3a* (*Hd3a*) and *RICE FLOWERING LOCUS T 1* (*RFT1*), *Zea mays Centroradialis 8* (*ZCN8*), and soybean Glyma.16G150700 (*GmFT2a*), Glyma.16G044100 (*GmFT5a*) regulating flowering, *FT* genes also participate in regulating various aspects of plant growth and development. For example, the potato (*Solanum tuberosum*) gene *SELF-PRUNING 6A* promotes tuber formation under SDs (Navarro et al., 2011). The homologue of *FT* in European aspen (*Populus tremula*), *FLOWERING LOCUS T2*, is involved in SD-induced growth stagnation and bud set (Ding and Nilsson, 2016). *SINGLE FLOWER TRUSS* (*SFT*) accelerates secondary cell wall biogenesis and promotes vascular maturation independently of photoperiodic flowering in tomato (*Solanum lycopersicum*; Shalit-Kaneh et al., 2019).

Notably, besides a floral activator (florigen), the PEBP family also includes floral inhibitors (coined anti-florigen) such as *TERMINAL FLOWER 1* (*TFL1*), *RICE CENTRORADIALIS* (*RCN*), and *SELF PRUNING* (*SP*; Higuchi, 2018). In rice, RCN inhibits flowering by competing with Hd3a for 14-3-3 binding to form a florigen repression complex (FRC), and the balance between FRC and FAC regulates the development of the SAM (Kaneko-Suzuki et al., 2018). The *SP* gene of tomato regulates plant architecture by inhibiting lateral reproductive bud growth. Plant architecture is shorter and more compact in the sp. mutant, and yield increases (Pnueli et al., 1998). The cotton (*Gossypium barbadense*) sp. mutant has altered branching architecture and produces clustered bolls (Si et al., 2018).

**Figure 1:**
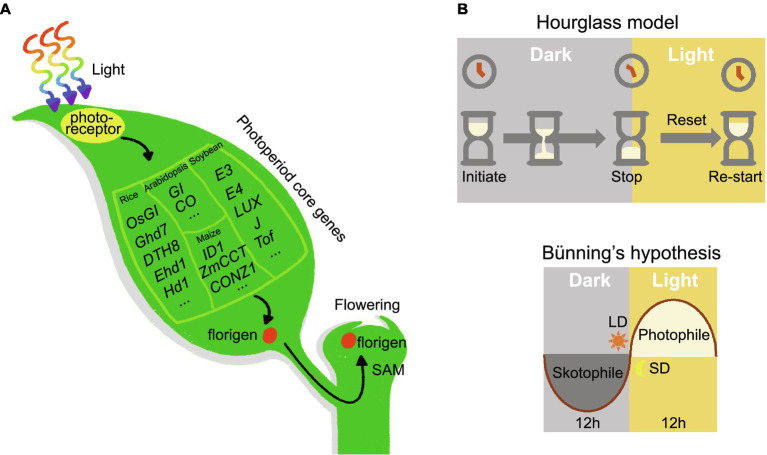
Universality of photoperiod flowering signaling pathway in plant. **(A)** Photoreceptors receive the input signal, the photoperiod core network transmits the signal, and the florigen enacts the output signal. **(B)** Hourglass hypothesis. Timekeeping begins at night and stops after a period of darkness. This model requires exposure to light to turn the hourglass over and restart timing. Bünning’s hypothesis. Photoperiodic timekeeping depends on the circadian clock. For short-day plants, darkness is required during the photophile (light-requiring) phase; long-day plants demand light exposure in the skotophile (dark-requiring) phase.

Moreover, florigen and anti-florigen contribute to plant heterosis. Tomato plants carrying mutations in *SFT* are characterized by extremely delayed flowering and few fruits, but plants heterozygous for *SFT* exhibit dramatically increased yield (Park et al., 2014). In addition, introducing different mutations in *SFT* into the sp. mutant background expands the plant body plan and increases yield in tomato (Park et al., 2014). In rice, the dosage of florigen alleles also affects heterosis (Huang et al., 2016). Rapeseed (*Brassica napus*) plants heterozygous for the mutant allele of *TERMINAL FLOWER 1* showed increased seed number per pod and total seeds per plant compared to wild-type plants (Guo et al., 2014). Since heterozygous plants cannot be maintained due to segregation in the progeny, molecular biology techniques may be employed to modify the florigen/anti-florigen genes *in situ* to produce florigen/anti-florigen pairs with different expression levels, thus helping to establish stable heterosis and improve crop yield (Eshed and Lippman, 2019). Given the versatility of florigen/anti-florigen genes, crop performance and quality can be improved by studying florigen/anti-florigen. Furthermore, plant responses to photoperiodic flowering are ultimately achieved by activating the expression of florigen (Figure 1A).

## Development and Present Understanding of the Photoperiodic Model

The discovery of photoperiodism inspired scientists to study how plants measure daylength. Initially, scientists proposed two hypotheses: the hourglass hypothesis and Bünning’s hypothesis (Figure 1B). The hourglass hypothesis states that photoperiod timing begins at night and stops after a critical length of darkness; it requires a period of exposure to light to restart the timer. Thanks to the discovery of phytochrome (see below), Sterling Hendricks proposed a more specific hourglass model in 1960 (Hendricks, 1960). Phytochrome is a red/far-red light photoreceptor that absorbs red light during the day in its inactive form (Pr), converting it to the active form (Pfr); at night, active Pfr reverts back to Pr (Li et al., 2011). The transition between Pr and Pfr states acts like an hourglass, and the Pr/Pfr ratio determines the plant photoperiodic response. Night breaks are equivalent to inverting the hourglass twice within a short time. Theoretically, two fast reversals in this situation should not have much influence on the time perceived by the hourglass. However, in practice a short illumination at night can inhibit the flowering of SDPs, disproving this hypothesis (Thomas and Vince-Prue, 1996).

The hypothesis introduced by the German scientist Erwin Bünning in 1936 posited that circadian rhythms are the basis for photoperiodic time measurements (Bunning, 1936). Circadian rhythms were predicted to be divided into two phases: the photophile phase, which needs light, and the skotophile phase, which requires darkness. During these two phases, the presence or absence of external light tells the plant whether it is exposed to short or long days. In the 1950s, Colin Pittendrigh extended Bünning’s hypothesis and proposed the external coincidence model, which states that the photoperiodic response is activated when light coincides with the endogenous circadian rhythm at a particular stage (Pittendrigh and Minis, 1964). In this model, light holds two functions: signal activation but also entrainment. In Pittendrigh (1966) realized that a mechanism relying on internal rhythms may also be used to explain the measurement of photoperiod (Pittendrigh, 1966). The internal coincidence model suggests that external signals [such as dawn (lights on) and dusk (lights off)] will produce two different rhythmic oscillations. The induction or repression of signaling is triggered only when these oscillations are synchronized. These two models are now widely accepted and have been validated in different plants.

**Figure 2:**
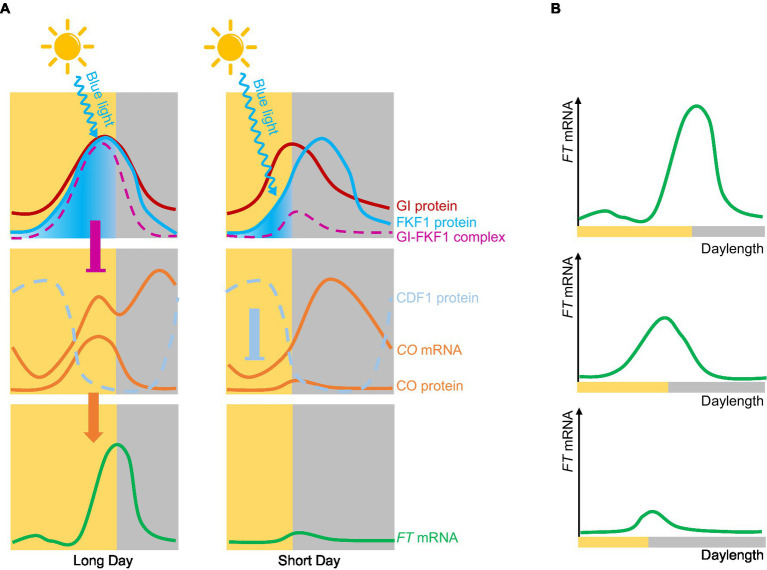
Internal and external coincidence models of photoperiodic flowering in Arabidopsis. **(A)** Molecular mechanism of Arabidopsis flowering. Under long-day conditions, the expression peaks of *GI* and FLAVIN-BINDING, KELCH REPEAT, F-BOX *1* (*FKF1*) are the same. FKF1 is activated by blue light and forms a complex with GI; this complex represses the expression of CYCLING DOF FACTOR 1 (*CDF1*), thereby promoting *CO* expression and further activating the expression of *FT.* Under short-day conditions, the expression peaks of *GI* and *FKF1* are out of synchrony, and only very low levels of the GI-FKF1 complex are formed, so that CDF1 accumulates and inhibits *CO* expression of *CO*, which in turns reduces *FT* expression. **(B)** *FT* mRNA gradually increases with longer daylengths.

Arabidopsis (*Arabidopsis thaliana*) is a typical LDP that perfectly fits the external coincidence model (Salazar et al., 2009). *CONSTANS* (*CO*) was the first photoperiod-related molecule to be cloned (Putterill et al., 1995). *CO* encodes a zinc finger transcription factor whose transcript levels are under the control of the circadian clock and daylength. Peak *CO* mRNA levels are observed from ZT12 (zeitgeber time 12, with dawn counting as ZT0) to dawn under LDs, or from ZT12 to ZT20 under SDs (Suarez-Lopez et al., 2001). The stability of CO is regulated by light, with far-red light and blue light stabilizing the CO protein, while red light promotes its degradation (Valverde et al., 2004). Thus, through both transcriptional and posttranslational regulation, CO forms an external coincidence sensor that activates the expression of its downstream target *FT* (Salazar et al., 2009). The blue light photoreceptor FLAVIN-BINDING, KELCH REPEAT, F-BOX *1* (FKF1) is another external coincidence detector; it contains a LOV domain, and the expression of its encoding gene is regulated by the circadian clock (Imaizumi et al., 2003). FKF1 is activated by blue light and regulates *CO* expression by degrading the transcriptional repressor CYCLING DOF FACTOR 1 (CDF1; Imaizumi et al., 2005). In Arabidopsis, the two external coincidence sensors CO and FKF1 support the external coincidence model. The circadian clock also regulates *GIGANTEA* (*GI*) expression and GI function to regulate photoperiodic flowering in Arabidopsis (Fowler et al., 1999). GI interacts with blue light-activated FKF1 to form a complex, the formation time of which is closely related to *CO* expression. Under LD conditions, the expression patterns of *FKF1* and *GI* are synchronized, peaking in expression in the afternoon. FKF1 is activated by light, leading to the formation of the FKF1-GI complex to degrade CDF1 and promote *CO* expression before dusk. Under SD conditions, the expression patterns of *FKF1* and *GI* are not synchronized, resulting in only the minute formation of the GI-FKF1 complex; CDF1 can then accumulate to repress *CO* expression (Sawa et al., 2007). The timing of formation of the FKF1-GI complex is regulated by both the external and internal coincidence model (Figure 2).

## Critical Daylength Sensing and Gradual Daylength Sensing

**Figure 3:**
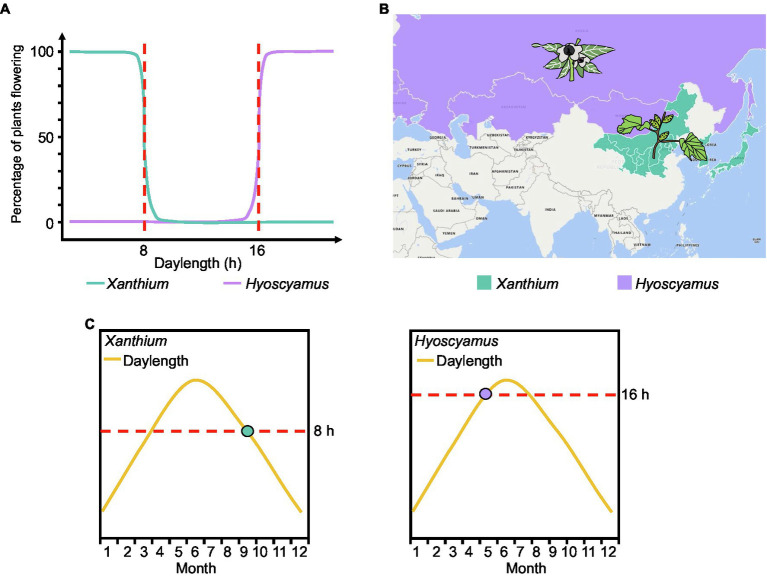
Traditional and new measurement methods of critical daylength. **(A)** As the flowering ratio of plants under different daylengths. The red dotted line represents the critical daylength. **(B)** Geographical distribution of *Xanthium* and *Hyoscyamus*. **(C)** Putative expression time point of the florigen genes in *Xanthium* and *Hyoscyamus*. The dots represent the point when the florigen begins to be expressed.

Early plant physiologists investigated flowering of plants exposed to different daylengths and expressed the results as a flowering ratio; they discovered that many plants appear to have a critical daylength below (for SDPs) or above (for LDPs) which flowering will occur. For example, *Xanthium strumarium* (a SDP) flowers when the daylength is shorter than 8h (critical daylength threshold=8h), while *Hyoscyamus niger* (a LDP) does not flower until daylength exceeds 16h (critical daylength threshold=16h; Figure 3A; Thomas and Vince-Prue, 1996). However, models based on existing expression data confirm that *FT* expression does not sense any critical threshold in daylength in Arabidopsis. Instead, *FT* transcript levels gradually increase with longer daylengths (Figure 2B).

**Figure 4:**
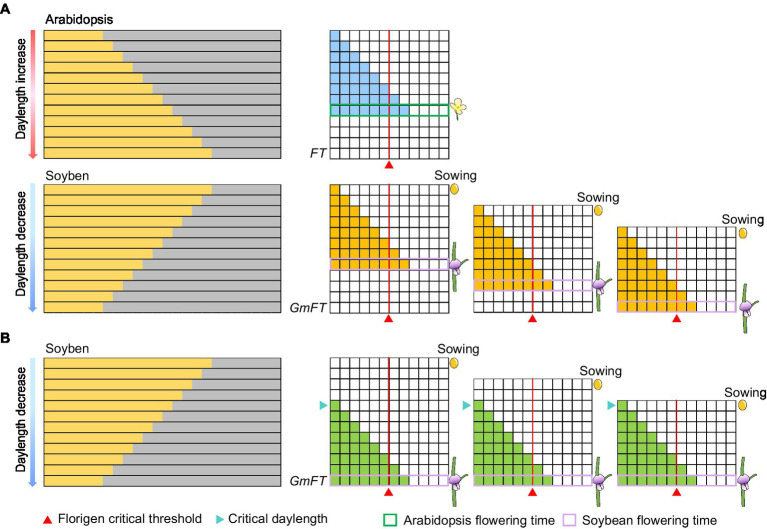
Expression patterns of florigen in different models. **(A)** Expression patterns of florigen change gradually with daylength. Florigen expression gradually increases with daylength; the plants flower when a critical threshold is reached. **(B)** Expression patterns of florigen with critical daylength threshold. The florigen genes begin to be expressed when the daylength is shorter than the critical daylength, resulting in flowering of soybean when the critical threshold is reached. The number of filled horizontal squares represents the mRNA levels of florigen. The red triangle represents the critical threshold of florigen. The cyan triangle represents the critical daylength.

In fact, Arabidopsis with the gradual daylength sensing as a model plant for photoperiod flowering study is not enough to explain the critical daylength sensing mechanism. Before photoperiodism was proposed, soybean farmers in the USA hoped to harvest soybeans in stages, but they discovered that soybean plants flower at almost the same time despite their different sowing times. By analogy to the theory that Arabidopsis perceives the gradual change in daylength, the expression of florigen in soybean was predicted to gradually increase as daylength shortens, thus inducing flowering when a certain threshold is reached. As the time for florigen to reach the critical threshold in soybean would be different, its flowering time would also be different (Figure 4A). However, results of simulations using gradual daylength sensing are inconsistent with the real-life observations of American soybean farmers (Figure 4A). Thus, the external coincidence model based on Arabidopsis cannot fully capture photoperiod perception in plants globally (Salazar et al., 2009; Itoh and Izawa, 2013). In the Japanese scientist Itoh et al. (2010) cleverly used the expression levels of the florigen gene to analyze the critical daylength threshold of rice to replace the flowering ratio. Recently, we followed a similar method to confirm that some soybean varieties can recognize critical daylength thresholds (Qiu et al., 2021). Here, we further explored critical daylength sensing to simulate flowering time in soybean sown at different times. When daylength during the early growth period is longer than the critical daylength threshold, the florigen genes are not expressed in earlier planted soybeans. By contrast, when daylength is shorter than the critical daylength in late summer, the florigen genes are expressed simultaneously in all soybeans regardless of sowing times (Figure 4B). As a result, soybeans planted in staggered stages can nonetheless flower simultaneously (Figure 4B). Obviously, the sensing of the critical daylength is not entirely beneficial to the production and application in soybean. Interestingly, mutations in the photoperiod gene *Grain number, plant height, and heading date* 7 (*Ghd7*) in rice can transform critical daylength sensing to gradual daylength sensing (Qiu et al., 2021). This strategy may be conducive to staggered sowing and harvesting for rice production.

## Daylength Sensing and Latitude Distribution

Daylength sensing is essential for plants to perceive the changing seasons and regulate flowering time accordingly (Itoh and Izawa, 2013). Here, we emphasize the overlooked phenomenon whereby daylength sensing directly determines the latitude distribution of plants. For example, the critical daylength sensing of *Xanthium* (SDP) is 8h (Figure 3A). In the northern hemisphere, daylength is less than 8h at high latitudes during autumn. Therefore, *Xanthium* is primarily distributed in Inner Mongolia, Korea, Japan, and other high-latitude regions and flowers when the daylength falls below 8h in the summer–autumn season (Figures 3B,C). Similarly, the critical daylength sensing of *Hyoscyamus* (LDP) is 16h (Figure 3A). Summer daylength will reach 16h at high latitudes. Therefore, *Hyoscyamus* is primarily distributed in high-latitude regions such as Mongolia, Russia, and Europe and flowers when daylength is over 16h in summer (Figures 3B,C). In nature, not all *Xanthium* and *Hyoscyamus* are distributed in high-latitude regions, and some varieties can flower at mid-latitude. This phenomenon may be associated with mutations in genes participating in photoperiodic perception.

*Heading date 1* (*Hd1*), the rice homologue to Arabidopsis *CO*, promotes flowering under SD conditions but represses flowering under LD conditions (Yano et al., 2000). *Early heading date 1* (*Ehd1*) encodes a B-type response regulator and inducing flowering by activating the transcription of *Hd3a* and *RFT1* (Doi et al., 2004). *Ghd7*, which encodes a protein with a CCT (CO, CO-like, and TOC1) domain, is expressed under LD conditions and was identified as a repressor of flowering (Xue et al., 2008). *Ghd8* [also named *Days to Heading on chromosome 8* (*DTH8*), *Heading date 5* (*Hd5*), and *Late Heading Date 1* (*LHD1*)] encodes a CCAAT-box binding factor that prevents flowering by repressing the expression of *Ehd1* and *Hd3a* in LD conditions (Wei et al., 2010; Yan et al., 2011; Dai et al., 2012; Fujino et al., 2013). *DTH7* [also named *Ghd7.1* and *PSEUDO-RESPONSE REGULATOR 37* (*OsPRR37*)] encodes a pseudo-response regulator protein that delays flowering by repressing *Hd3a* expression under LD conditions (Koo et al., 2013; Liu et al., 2013; Gao et al., 2014). Currently, we demonstrated that different combinations of rice photoperiod-related genes in rice varieties directly determine their latitude distribution according to changes in daylength sensing (Qiu et al., 2021). The allelic combinations at *Ghd7*, *Hd1*, and *DTH8* play an essential role in the regulation of rice daylength sensing but also control rice distribution at different latitudes (Qiu et al., 2021). Thus, we speculated that the differences between the origins of crops and their current planting areas indicate that crops often experience changes in daylength sensing during domestication, which may include a series of polymorphisms in photoperiod genes. For example, soybean originated from mid-latitude regions but now grow at all latitudes. The genes *J*, *Time of Flowering 11* (*Tof11*), and *Tof12* participate in the photoperiod response of soybean and promote the expansion of soybean to high- and low-latitude regions (Lu et al., 2017, 2020). In rice, natural variation at the flowering promoting factors *Days to heading on chromosome 2* (*DTH2*) and *Early heading date 4* (*Ehd4*) plays an important role in the northward expansion of rice cultivation range (Gao et al., 2013; Wu et al., 2013). Natural variation of the flowering inhibitors *Ghd7*, *DTH8,* and *DTH7* reduces rice photoperiod sensitivity and is contributing to an expansion of rice growing regions to more temperate zones and colder zones (Xue et al., 2008; Fujino et al., 2013; Koo et al., 2013). Due to the bifunctionality of *Hd1* mentioned above, rice varieties carrying *hd1* mutants were identified at different latitudes (Takahashi and Shimamoto, 2011). The freight transport system was underdeveloped during the early stages of human social development. The ability to adapt to a wide range of latitudes is crucial for the latitudinal expansion of plants, especially crops. Only plants that adapted to the various light environments at different latitudes were widely planted and ultimately became a primary food source and cash crops. Therefore, the agronomic application of plant photoperiodic flowering is of great significance to the genetic improvement of major crops. Through molecular biology methods, combined with the photoperiod conditions of a given growth region, the optimal combination of photoperiodic genes may be selected in a more targeted and effective manner.

## A System for Daylength Sensing Based on Florigen Gene Expression

Rice is also a typical plant with a critical daylength, and molecular genetic analysis over the last 20years has revealed its underlying genetic network. The photoperiodic flowering network of rice comprises multiple signaling pathways, including OsGI-Hd1-Hd3a, OsGI-Ehd1, EARLY FLOWERING 3-1 (OsELF3-1)-Ghd7-Ehd1-Hd3a, and DTH8-Ehd1-Hd3a (Hayama et al., 2003; Doi et al., 2004; Xue et al., 2008; Wei et al., 2010; Zhao et al., 2012). These signaling pathways ultimately converge on the expression of *Hd3a* and *Ehd1*, which is regulated by the DTH8-Hd1 and Ghd7-Hd1 protein complexes (Du et al., 2017; Zhang et al., 2017). In addition, the rice genome harbors two homologous genes for most genes of the photoperiodic flowering signal network. For example, *RFT1* (also named *FT-L3*) is a paralogue of *Hd3a* (Komiya et al., 2008, 2009), and *Hd1* is a paralogue of *DTH2* (Wu et al., 2013). The presence of these paralogous genes enhances the stability of the entire signaling network. In recent years, several groups have demonstrated that Hd1, DTH8, Ghd7, and DTH7 can interact with each other to form Hd1-DTH8-Ghd7-DTH7 modules that regulate photoperiodic flowering (Nemoto et al., 2016; Du et al., 2017; Zhang et al., 2017; Zong et al., 2020).

**Figure 5:**
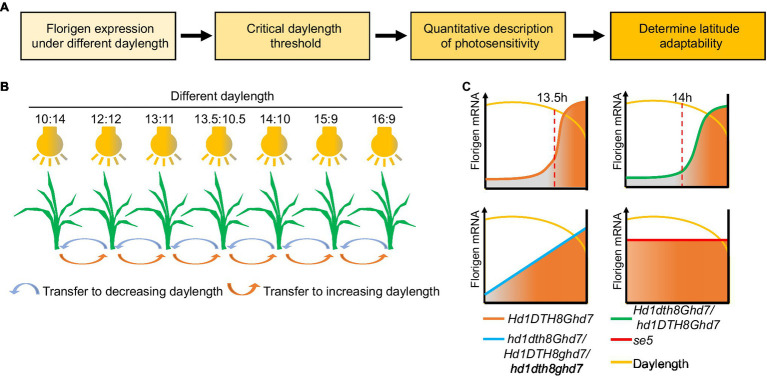
Process and application of the daylength recognition model based on florigen. **(A)** Flowchart of the daylength-sensing-based environment adaptation simulator (DEAS) system. **(B)** Schematic diagram of the DEAS system. Plants are sown and grown in greenhouses under different photoperiods. After a given number of days, plants are transferred to the next shorter or longer daylength in the series to simulate daylength changes in nature. By detecting the expression of florigen genes under different daylengths, the critical daylength threshold can be obtained. **(C)** Critical daylength sensing and florigen expression changes in different rice genotypes. When daylength is shorter than the critical daylength, the florigen gene is highly expressed. Orange line, cultivars with original critical daylength sensing; green line, cultivars with fine-tuned daylength sensing; blue line, cultivars with gradual daylength sensing; and red line, cultivars with daylength insensitivity.

In Itoh et al. (2010) showed that two distinct gating mechanisms (the flowering promoter Ehd1 is regulated by blue-light gating; the flowering repressor Ghd7 is regulated by red-light gating) play a key role in measuring critical daylength in rice, whose threshold is 13.5h (Itoh et al., 2010; Itoh and Izawa, 2013). Based on Izawa’s method, we recently established a daylength-sensing system using multiple intelligent climate chambers to simulate different daylength conditions (Figure 5). Florigen gene expression data were collected from rice seedlings exposed to the various daylengths, which can then be used to determine the daylength sensing process of different rice cultivars. Variation in the core photoperiod genes *DTH8*, *Hd1*, *Ghd7*, and *DTH7* can modulate daylength sensing in rice (Qiu et al., 2021). Indeed, *DTH8Hd1Ghd7DTH7* can sense the original critical daylength threshold but often do not exhibit wide adaptability in breeding. However, mutations in *DTH8*, *Hd1*, *Ghd7*, or *DTH7* can change the original critical daylength sensing to different daylength sensing methods for adaptation to different latitudes. For example, at high latitudes, *ghd7* alleles in *japonica* cultivars convert plants from sensing critical daylength to perceiving gradual daylength, thereby promoting early heading of these varieties and escaping the low temperature of late autumn typical of these high-latitude environments to achieve high yield. Among the hybrid *indica* cultivars, the three-line hybrid rice system mainly uses the *dth8* allele to fine-tune critical daylength threshold, enhancing latitudinal adaptation, while two-line hybrid rice uses the *hd1* allele (Qiu et al., 2021).

## Prospects for the Applications of the Daylength-Sensing System

Although the daylength-sensing system cannot directly explain the molecular mechanism of daylength-sensing in plants, we believe this system can enrich the research methods implemented for photoperiodic flowering: The daylength-sensing system as a “calendar” provides information on a plant’s sensing season. In addition, the daylength-sensing system as a “location system” provides information on a plant’s sensing latitude. First, the model can be used to compare the response to reference varieties from different latitudes and to characterize their daylength-sensing properties at different simulated latitudes. Second, the model can help classify breeding materials and populations according to reference varieties for subsequent breeding. Third, for new crops from which functional photoperiod genes have not been cloned, or for some crops (like soybean and maize) from which functional photoperiod genes have been cloned, the model can offer a predictive role by measuring the expression of florigen genes to accelerate the selection of adapted germplasm.

The ability of crops to adapt to the latitude in a specific ecological environment ensures that the growth period of crops is aligned with the local growing season so that maximum yield can be achieved. Latitude adaptation is essential for crop breeding, the introduction of new varieties, hybrid seed production, and yield. However, the improvement of crop latitude adaptation by adjusting the growth period in traditional breeding is a lengthy process. Global climate change will affect crop yields and threaten food security by increasing the frequency and intensity of extreme heat, drought, and waterlogging events. We will need to exploit germplasm resources resistant to these stresses to improve varieties and mitigate the negative effects of climate change on yield. At the same time, rising temperatures at high latitudes may provide an opportunity for crops to expand their growing regions into higher latitudes and extend the growing season of summer crops. The planting structure of crops should be adjusted according to the cultivation environment. In agricultural production, crop rotation is mainly used to increase land utilization and increase crop production (Xu et al., 2021). The main types of crop rotations include cereal–legume rotations and legume–grass rotations. Adjusting the rotation patterns of existing crops will be more conducive to maximizing yield. These processes all require accelerated selection methods for crop latitude adaptation. The daylength-sensing system presented here predicts the adaptability of a plant to latitude by estimating their critical daylength. The implementation of molecular biology methods (such as CRISPR/Cas9-mediated genome editing of photoperiod genes) will accelerate the generation of improved crops better adapted to latitude. Notably, the daylength-sensing system provides an efficient and accurate pipeline for crop selection to latitude adaptation.

The photoperiod response of plants is universal: Photoreceptors perceive input signals (daylength), photoperiod networks transmit these signals, and florigen outputs a response (Figure 1A). However, the specific networks are not identical across plants. For example, soybean uses the *J* and *LUX ARRYTHMO*, which are not typically used in rice (Matsubara et al., 2012; Saito et al., 2012; Yang et al., 2012; Zhao et al., 2012). Maize exhibits genetic diversity in the promoter regions of *ZmCCT* and *ZCN8* to regulate latitudinal adaptation (Guo et al., 2018). Rather than focusing on these differing components, the daylength-sensing system directly measures the expression of florigen genes, thereby capitalizing on the larger universal features of the photoperiod responses. We also demonstrated that this system successfully detects daylength sensing in soybean and maize varieties (Qiu et al., 2021). Therefore, the daylength-sensing system also provides a universal method for the selection of crops adapted to latitude.

Crop adaptation to latitude is regulated by daylength sensing and other environmental factors, including ambient temperature. The current version of the daylength-sensing system can only account for daylength. With global warming, temperatures will increase over current levels at different latitudes (Allen et al., 2018; Cox et al., 2020). The basic helix–loop–helix transcription factor PHYTOCHROME INTERACTING FACTOR 4 (PIF4) activates the expression of *FT* at high temperatures in Arabidopsis (Kumar et al., 2012). Studies have shown that warm night temperatures severely affect crop growth and yield (Jagadish et al., 2015). Due to growth season—long rises in temperatures, the yield of several rice cultivars has decreased in central and southern regions of China (Yang et al., 2017). Simulating warm night temperatures in the field using a field-based infrared ceramic heating system caused abnormal expression of circadian clock genes, resulting in a decrease in rice yield (Desai et al., 2021). High temperatures lower the expression levels of *Ghd7* and increase the expression of *RFT1*, causing early flowering (Nagalla et al., 2021). Therefore, we plan a number of follow-up studies on the molecular mechanisms of temperature sensitivity to help improve the daylength-sensing system in the future. Other environmental factors like light quality, sugar, and various stress conditions can also be incorporated in growth chambers to improve the daylength-sensing system, which will be instrumental for studying the interaction between photoperiod and climate change.

## Author Contributions

XW, PZ, RH, JZ, and XO wrote the paper. All authors contributed to the article and approved the submitted version.

## Funding

This work was supported by the National Key R&D Program of China (2017YFA0506100).

## Conflict of Interest

The authors declare that the research was conducted in the absence of any commercial or financial relationships that could be construed as a potential conflict of interest.

## Publisher’s Note

All claims expressed in this article are solely those of the authors and do not necessarily represent those of their affiliated organizations, or those of the publisher, the editors and the reviewers. Any product that may be evaluated in this article, or claim that may be made by its manufacturer, is not guaranteed or endorsed by the publisher.
